# Distinct Transmission Cycles of *Leishmania tropica* in 2 Adjacent Foci, Northern Israel

**DOI:** 10.3201/eid1212.060497

**Published:** 2006-12

**Authors:** Milena Svobodova, Jan Votypka, Jitka Peckova, Vít Dvorak, Abedelmajeed Nasereddin, Gad Baneth, Julia Sztern, Vasiliy Kravchenko, Amnon Orr, David Meir, Lionel F. Schnur, Petr Volf, Alon Warburg

**Affiliations:** * Charles University, Prague, Czech Republic;; † The Hebrew University of Jerusalem, Jerusalem, Israel;; ‡ Tiberias Veterinary Center, Tiberias, Israel;; § Nature and National Parks Protection Authority, Jerusalem, Israel

**Keywords:** Cutaneous leishmaniasis, rock hyrax, Procavia capensis, Leishmania tropica, Phlebotomus sergenti, Phlebotomus arabicus, zoonosis, sand flies, Israel, research

## Abstract

Transmission of Leishmania tropica was studied in 2 adjacent foci in Israel where vector populations differ. Only Phlebotomus sergenti was found infected with L. tropica in the southern focus; P. arabicus was the main vector in the northern focus. Rock hyraxes (Procavia capensis) were incriminated as reservoir hosts in both foci. L. tropica strains from the northern focus isolated from sand flies, cutaneous leishmaniasis cases, and rock hyraxes were antigenically similar to L. major, and strains from the southern focus were typically L. tropica. Laboratory studies showed that P. arabicus is a competent vector of L. tropica, and P. sergenti is essentially refractory to L. tropica from the northern focus. Susceptibility of P. arabicus may be mediated by O glycoproteins on the luminal surface of its midgut. The 2 foci differ with respect to parasites and vectors, but increasing peridomestic rock hyrax populations are probably responsible for emergence of cutaneous leishmaniasis in both foci.

## Abstract 2

TOC summary for table of contents: Infection with *Leishmania tropica* is emerging because of encroachment of rock hyraxes and transmission by multiple vector species.

Leishmaniases are parasitic diseases with a wide range of clinical symptoms and currently threaten 350 million persons in 88 countries (*1*). In Israel and its vicinity, Leishmania major and L. tropica cause cutaneous leishmaniasis (CL), and L. infantum can result in visceral leishmaniasis (*2*). Until recently, relatively little information was available on the epidemiology of CL caused by L. tropica in this region. Outbreaks were not investigated, and cases were usually grouped together with CL cases caused by L. major (*3*). However, in recent years, new foci of CL caused by L. tropica are emerging in different parts of the country, such as the Galilee region of northern Israel and the Judean Desert east of Jerusalem that warrant thorough investigations (*4**,**5*). Clinically, lesions caused by L. tropica last longer and are more difficult to treat than those caused by L. major (*6*). Although L. tropica can be anthroponotic, foci in Israel appear to be zoonotic, with rock hyraxes (Procavia capensis) serving as probable reservoir hosts (*4*).

Leishmania development in sand flies is facilitated by interaction with midgut molecules of the vector. Laboratory studies showed that sand flies are composed of 2 groups. Species such as Phlebotomus (Phlebotomus) papatasi, the vector of L. major and P. (Paraphlebotomus) sergenti, the main vector of L. tropica, show specificity for Leishmania they transmit in nature (*7**,**8*). Conversely, species such as Lutzomyia longipalpis, the vector of L. infantum in South America, and many others are permissive and support development of several Leishmania spp (*8**,**9*).

Studies performed with L. major and P. papatasi showed that attachment in the midgut is mediated by the major surface glycoconjugate of promastigotes, lipophosphoglycan (LPG), which interacts with PpGalec, a galactose-binding molecule in the midgut of P. papatasi (*10*). However, the mechanism of attachment may be redundant, and another molecule on the promastigote flagellum may be involved (*11*).

Recently, the susceptibility of phlebotomine sand flies to Leishmania parasites was shown to correlate with O-linked glycoproteins in sand fly midgut (P. Volf, unpub. data). The permissive species have O-glycosylated epitopes on the luminal midgut surface, which may serve as binding sites for lectinlike components found on the surface of parasites (*12**,**13*). We compare midgut glycosylation patterns of 2 sand fly species, P. (Adlerius) arabicus and P. (Paraphlebotomus) sergenti that transmit L. tropica in 2 adjacent foci in the Galilee region of northern Israel.

L. tropica is genetically heterogeneous, and strains are readily distinguishable by antigenic, biochemical, and molecular techniques (*14**–**16*). We report findings of extensive studies in 2 adjacent CL foci that demonstrate conclusively that both vector species and parasite strains from the northern focus are different from those in the southern focus, a mere 10 km away (Figure 1).

**Figure 1:**
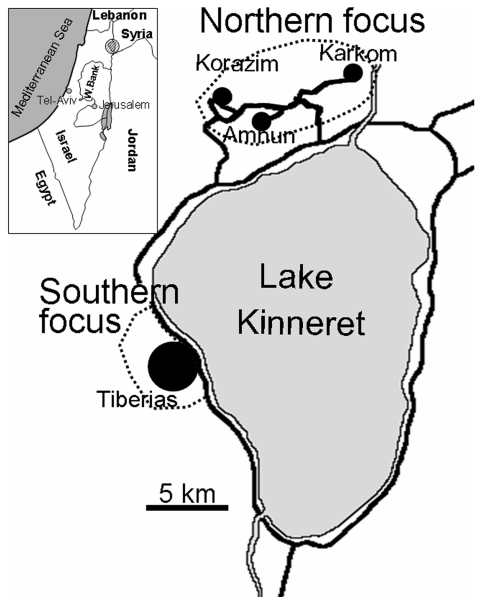
Leishmania tropica foci near Lake Kinneret in the Galilee region of Israel. Inset shows the location of the foci. W. Bank, West Bank.

## Materials and Methods

### Study Area

Studies were conducted in 2 adjacent foci in the Galilee region of northern Israel (Figure 1). The northern focus comprises several villages situated on generally south-facing slopes ≈5 km north of Lake Kinneret in the eastern lower Galilee of northern Israel (32°55´N, 35°36´W). The area investigated encompasses the villages of Amnun (at sea level), Karkom (100–150 m above sea level), and Korazim (150 m above sea level), which have ≈1,200 inhabitants living in ≈300 single-family houses surrounded by gardens and built on basalt rock. Many boulders from the cleared land have been piled into large heaps separating individual plots and surrounding the villages. These boulder mounds are inhabited by numerous rock hyraxes (P. capensis).

The southern focus includes the city of Tiberias (32°47´N, 35°32´W; population = 38,952). Studies were conducted in the outskirts of urban neighborhoods, where boulder mounds were inhabited by large populations of rock hyraxes. These neighborhoods are built on north- to northeast-facing slopes.

### Collection, Dissection, and Identification of Sand Flies

Sand flies were trapped by using CDC light traps (John W. Hock, Gainesville, FL, USA) in September 2002 and 2004. Dead flies were stored in 70% alcohol and identified by using several keys (*17**–**19*). Live female flies were immobilized on ice, rinsed briefly in 96% ethanol, and dissected in 0.9% sterile saline. Guts were microscopically examined for parasites. Heads and genitalia were used for identification. Guts containing promastigotes were aseptically placed in glass vials (2.5 mL) containing blood agar made from defibrinated rabbit blood overlaid with a 1:1 mixture of RPMI 1640 medium and Schneider Drosophila cell culture medium supplemented with 10% fetal calf serum (Sigma, Saint Louis, MO, USA, and Gibco-BRL, Gaithersburg, MD, USA), 10,000 IU penicillin (Biotika, L'upca, Slovakia), 100 μg/mL amikacin (Bristol-Myers Squibb, Princeton, NJ, USA), and 1,500 μg/mL 5-fluorocytosine (Sigma). Some data on Leishmania isolates from the northern focus were obtained from our previously published results.

### Collection of Animals

Rock hyraxes were trapped by using raccoon traps (http://www.havahart.com) baited with fresh leaves and anesthetized with ketamine (10 mg/kg given intramuscularly). Samples of blood and skin were obtained for parasite culture and blotted onto filter paper for PCR analysis. Animals were released at the site of capture. Skin biopsy specimens were homogenized and placed in blood agar culture medium in flat tubes (Nunclon; Nunc Nalgene International, Rochester, NY, USA). Rats (Rattus rattus) were trapped by using steel mesh traps (Tomahawk Live Trap Co., Tomahawk, WI, USA) placed in sewers and rock crevices. Spiny mice (Acomys cahirinus) were captured by using Sherman traps (H.B. Sherman Traps, Tallahassee, FL, USA). Rodents were anaesthetized with ketamine/xylazine (150 mg/kg and 15 mg/kg, respectively, given intraperitoneally). Blood from the tip of the tail was blotted on filter paper. Ear biopsy specimens were treated as described for hyraxes. Cultures were checked at 4–7-day intervals for 1 month.

### DNA Extraction

DNA from wild-caught sand flies kept frozen or preserved in 100% ethanol was extracted as previously described (*20*). DNA from filter paper disks was extracted by using the phenol-chloroform method (*21*).

### Detection and Identification of Leishmania infections by PCR

The ribosomal internal transcribed spacer region 1 (ITS1) was amplified with Leishmania-specific primers. ITS1 PCR products showing a Leishmania-specific band on agarose gels were digested with HaeIII for species identification (*22*). Restriction fragments were subjected to electrophoresis on agarose gels and compared with DNA of L. infantum (Li-L699), L. major (Lm-L777), and L. tropica (Lt-L590).

### Antigenic Characterization of Parasite Isolates

Initial screening of isolates was performed by using gel diffusion of glycoconjugates secreted into culture media (excreted factor) and several antileishmanial serum samples (*23*). Leishmania-specific monoclonal antibodies (MAbs) were used in indirect immunofluorescent antibody (IFA) assays to determine surface antigenic characteristics of parasites (14). Briefly, promastigotes from primary cultures of new isolates and controls of L. infantum (Li-L699), L. major (Lm-L777), and L. tropica (Lt-L590) were placed in wells of fluorescent antibody slides (Bellco Glass Inc., Vineland, NJ, USA), dried, and fixed in cold acetone. Slides were blocked with 5% fetal bovine serum in phosphate-buffered saline (PBS) for 1 hour at room temperature. Mouse MAbs specific for L. major (T1), L. tropica (T11, T14, and T15), L. tropica/L. major (T3), and L. infantum/L. donovani (D2) were applied for 1 hour at 37°C. Goat anti-mouse immunoglobulin G conjugated with fluorescein isothiocyanate was applied for 40 minutes at 37°C in the dark. The preparations were washed 3 times with PBS plus 5% Tween 20 between incubations. Slides were mounted in 3% DABCO (Sigma) in PBS/glycerol and viewed with an Axiovert microscope (Zeiss, Göttingen, Germany).

### Experimental Infection of Sand Flies

Laboratory colonies of P. sergenti and P. arabicus were established from gravid females caught in the northern focus. The colonies were maintained at 23°C–25°C, 100% humidity, and 14:10 light:dark photoperiod. Adults had access to cotton wool soaked in 50% honey. Females were allowed to feed twice a week on mice anaesthetized with a ketamine/xylazine mixture (150 mg/kg and 15 mg/kg). Fed females were placed in plaster of paris–lined oviposition containers, and larvae were maintained on a decaying rabbit feces/rabbit chow mixture (*24*). Sand flies were infected by membrane feeding on heat-inactivated rabbit blood containing 5×10^5^ promastigotes/mL. Fed females were maintained at 23°C and dissected on day 9 after feeding, when infections were mature. Guts were checked microscopically for Leishmania promastigotes. Infection intensity was scored as light (<50 promastigotes/gut), moderate (50–500 promastigotes/gut), and heavy (>500 promastigotes/gut). L. tropica strains from the northern (IARA/IL/2001/L810, Amnunfly1) and southern (MHOM/IL/2001/L-836, Tiberias) foci were used for comparing susceptibility of sand flies to local strains. Promastigotes from the same culture and sand flies from the same batch were used in individual experiments. For every combination, the experiment was repeated twice. Statistical tests were performed by using Statgraphics version 4.2 software (StatPoint, Englewood Cliffs, NJ, USA).

### Glycosylation of Sand Fly Midguts

Midguts were dissected from 5- to 10-day-old P. sergenti and P. arabicus females. Midgut proteins were separated by sodium dodecyl sulfate–polyacrylamide gel electrophoresis on 10% gels under reducing conditions in a Mini-Protean III apparatus (Bio-Rad Laboratories, Hercules, CA, USA) at 200 V. Gels were stained with Coomassie brilliant blue R-250 or transferred to nitrocellulose membranes by using a Semiphor unit (Hoefer Scientific Instruments, San Francisco, CA, USA). Western blotting was performed for 90 minutes at 1.5 mA/cm^2^. Membranes were incubated with 20 mmol/L Tris, 150 mmol/L NaCl, 0.05% Tween (TBS-Tw) with 5% bovine serum albumin for 2 hours and then with Helix pomatia agglutinin (HPA) biotinylated lectin, which recognizes N-acetyl-d-galactosamine (GalNAc), a typical carbohydrate in O-glycans. In the control groups, HPA reactions were competitively inhibited by preincubation with 250 mmol/L GalNAc for 30 minutes. After repeated washing in TBS-Tw, blots were incubated for 1 hour with streptavidin peroxidase in TBS-Tw. The peroxidase reaction was developed with the substrate 4-chloro-1-naphthol. All chemicals for lectin blotting were obtained from Sigma.

### Random Amplified Polymorphic DNA Analysis

Twenty wild-caught sand flies morphologically identified as P. sergenti, 10 from the northern focus and 10 from the southern focus, were included in the analysis. Two flies from Tulek, Turkey, were included as an outgroup. DNA from thoraxes was extracted by using the High Pure PCR template preparation kit (Roche, Paris, France). Five decamer random primers (OPD5, OPE4, OPI1, OPI14, and OPI18; Operon Technologies Inc, Alameda, CA, USA) were used. The reaction mixture contained 12.5 μL master mixture (75 mmol/L Tris-HCl, pH 8.8, 20 mmol/L (NH_4_)_2_SO_4_, 0.001% Tween 20, 800 μM deoxynucleotide triphosphate mixture), 2.5 U Taq polymerase, 1.5 mmol/L MgCl_2_, 2 μL primer (10 pmol), and 8 μL double-distilled water in a final volume of 25 μL. Random amplified polymorphic DNA (RAPD) reactions were performed in a PTC-200 thermocycler (MJ Research Inc., Waltham, MA, USA) and subjected to 45 amplification cycles. PCR products were separated by electrophoresis on a 2% agarose gel in Tris-acetate EDTA buffer at 80 V for 3 hours and stained with ethidium bromide.

### ITS2 Sequencing

DNA samples for RAPD analysis were used for ITS2 sequencing. One specimen from each study area was included as previously described (*25*).

## Results

### Sand Fly Species

A total of 1,491 sand flies (7 species, 4 subgenera) from the northern focus and 876 sand flies (7 species, 4 subgenera) from the southern focus were identified. Phlebotomine fauna in the southern focus were relatively species poor with P. (Paraphlebotomus) sergenti comprising >90% of the flies. The most striking difference in the species composition between the foci was the absence of P. (Adlerius) arabicus and P. (Adlerius) simici from the southern focus, both of which were prominent species in the south-facing slopes of the northern focus (Table 1) (*26*).

**Table 1:** *Phlebotomus* sand fly species in the Galilee foci, northern Israel*

Species	Northern focus		Southern focus	
	No. (%) females	No. (%) males	No. (%) females	No. (%) males
*P.* (*Adlerius*) *arabicus*	62 (15)	234 (22)	–	–
*P.* (*Adlerius*) *simici*	35 (9)	118 (11)	–	–
*P.* (*Paraphlebotomus*) *sergenti*	131 (32)	317 (29)	267 (91)	532 (92)
*P.* (*Laroussius*) *tobbi*	167 (40)	337 (31)	11 (4)	23 (4)
*P.* (*Laroussius*) *syriacus*	–	12 (1)	8 (3)	16 (2)
*P.* (*Laroussius*) *perfiliewi*	10 (2)	31 (3)	1 (<1)	2 (<1)
*P.* (*Phlebotomus*) *papatasi*	9 (2)	28 (3)	5 (2)	7 (1)
Total	414	1,077	292	580

*Species comprising <1% of the fauna (1 *P*. [*Paraphlebotomus*] *alexandri* and 3 *P.* [*Adlerius*] *halepensis*) found in the southern focus were not included.

### Leishmania Infections in Sand Flies

To detect infections and obtain parasite isolates, sand fly females were dissected in sterile saline and guts were examined microscopically. Four (6.6%) of 61 P. arabicus and 1 (0.8%) of 125 P. sergenti from the northern focus had promastigotes in their guts. Infection intensity in P. sergenti from the northern focus was low, but all P. arabicus had heavy, mature infections. A total of 213 flies from the southern focus were dissected; 196 were P. sergenti, and 19 (9.7%) had promastigotes. Eleven of these females had heavy infections, and 8 had moderate-to-light infections. All infected females were caught at 1 sublocality in the southern focus, where the local infection rate was 19.6%. Promastigote cultures were established from 4 P. arabicus and 1 P. sergenti captured in the northern focus and from 18 P. sergenti females captured in the southern focus (Table 2). None of the other sand fly species were infected.

**Table 2:** *Leishmania tropica* infection rates among *Phlebotomus* sand flies and rock hyraxes in the Galilee foci, northern Israel*

Focus	Sand flies, rate (%)		Rock hyraxes, rate (%)
	*P. arabicus*	*P. sergenti*	
Northern	4/61 (7)†	1/125 (1)†	8/73 (11)†
Southern	Species not found	19/196 (10)†	6/46 (13)‡

**Figure 2:**
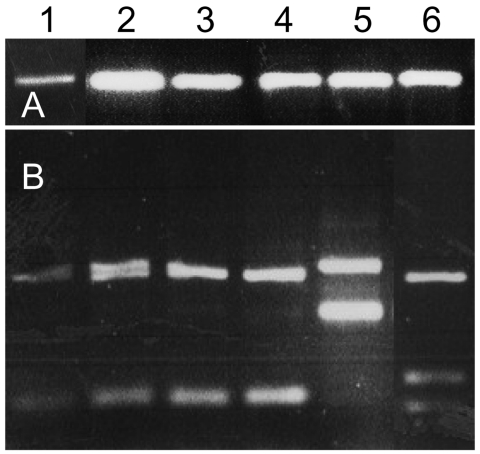
A) PCR of Leishmania internal transcribed spacer region 1 (ITS1) of naturally infected Phlebotomus sergenti sand flies and cultured Leishmania spp. controls. B) HaeIII digestion of restriction fragment length polymorphisms of ITS1 PCR products shown in A. Lane 1, P. sergenti female 1; lane 2, P. sergenti female 2; lane 3, P. sergenti female 3; lane 4, L. tropica (Lt-L590); lane 5, L. major (Lm-L777); lane 6, L. infantum (Li-L699).

**Figure 3:**
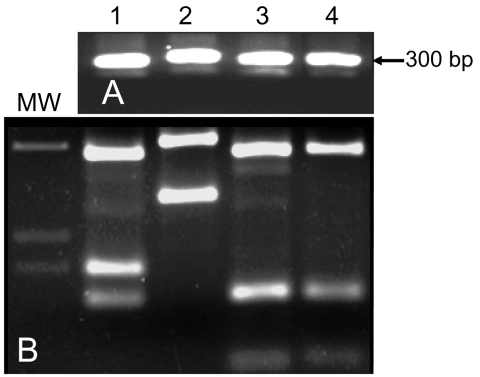
A) PCR of Leishmania internal transcribed spacer region 1 (ITS1) of cultured Leishmania promastigotes isolated from rock hyrax. B) HaeIII digestion of restriction fragment length polymorphisms of ITS1 PCR products shown in A. Lane MW, molecular mass marker; lane 1, L. infantum (Li-L699); lane 2, L. major (Lm-L777); lane 3, L. tropica (Lt-L590); lane 4, rock hyrax.

*Values are no. infected/no. tested (%). Sand fly infection rates were based on parasite isolation. Rock hyrax infection rates were determined by PCR and 1 isolate. †*Leishmania* species confirmed by internal transcribed spacer region 1 PCR and restriction fragment length polymorphism (Figure 2 and Figure 3). ‡*Leishmania* species confirmed by reverse line blot (data not shown).

Sand flies that were not dissected fresh were kept frozen and were subjected to ITS1 PCR for detection of Leishmania. Nine (18%) of 50 P. sergenti females from the southern focus were positive for Leishmania ribosomal DNA. HaeIII digestion of the ITS1 PCR products confirmed that all P. sergenti had L. tropica (Figure 2).

### Identification of Infections in Mammals

Rodents collected in the northern focus were tested for L. tropica infection by ITS1 PCR. Dried blood and skin samples from 28 rats (R. rattus) and 46 spiny mice (A. cahirinus) were negative for Leishmania DNA. Eight of 73 rock hyraxes from the northern focus and 6 of 46 rock hyraxes from the southern focus were positive for L. tropica DNA by ITS-1 amplification and reverse-line blotting using sequence-specific probes (data not shown). Of the positive animals, 11 were adults (9 females and 2 males) and 1 was a juvenile male. Parasites from 1 rock hyrax captured in the northern focus were cultured and identified by ITS1 PCR and digestion with HaeIII (Figure 3).

### Antigenic Characterization of Leishmania Isolates

IFA assays with species-specific MAbs were used to characterize different isolates. L. tropica isolates from the northern focus were antigenically distinct from all other isolates, including those from the southern focus (Table 3).

**Table 3:** Characterization of *Leishmania tropica* isolates from the Galilee foci, northern Israel*

Focus/ source	Monoclonal antibody specificity			Excreted factor serotype
	*L. major* T1	*L. major/L. tropica* T3	*L. tropica* T11	
Northern				
*Phlebotomus arabicus*	5+	5+	±	A4
Girl with CL†	4+	5+	–	A4
Rock hyrax	5+	5+	±	A4
Southern				
*P. sergenti*	–	4+	3+	A9B2
Man with CL†	–	2+	4+	A9B2
Reference strains				
*L. major*	5+	5+	–	A1
*L. tropica*	±	3+	3+	A9

*Characterization was performed by using excreted factor serotyping (*23*) and species-specific monoclonal antibodies (*14*). CL, cutaneous leishmaniasis. Values indicate relative intensity of fluorescence under UV light. *L. tropica* isolates from the northern focus were antigenically similar to *L. major* and distinct from other *L. tropica* strains. †Specimens were isolated by skin scraping for diagnostic purposes at the Department of Dermatology at Hadassah Hospital, Jerusalem.

### Susceptibility of P. arabicus and P. sergenti to L. tropica

**Figure 4:**
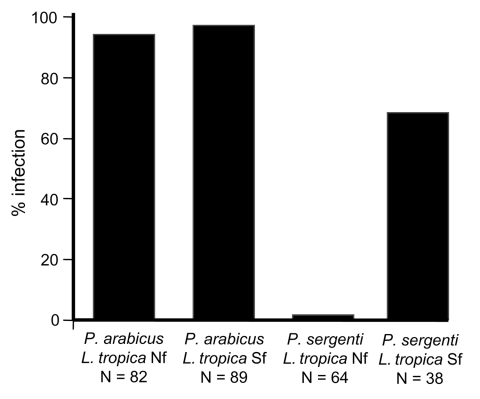
Artificial infection of laboratory-reared Phlebotomus arabicus and P. sergenti with Leishmania tropica isolates from 2 foci in Galilee, Israel. Note the high susceptibility of P. arabicus for both strains and refractoriness of P. sergenti for the northern strain. Nf, northern focus; Sf, southern focus.

In laboratory experiments, L. tropica parasites from the northern focus infected only P. arabicus, and parasites from the southern focus infected both P. arabicus and P. sergenti. Susceptibility of P. arabicus for infection with L. tropica strains from both northern and southern foci was high (94% and 97%, respectively). In contrast, P. sergenti was not permissive for L. tropica strains from the northern focus (1 of 64 flies). Susceptibility of P. sergenti for infection with L. tropica from the southern focus strain was lower (66%) than that of P. arabicus (Figure 4).

### Glycosylation of Luminal Midgut Proteins

**Figure 5:**
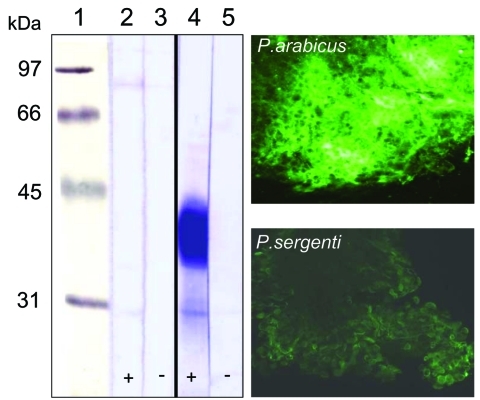
Left, female sand fly midgut lysates separated by sodium dodecyl sulfate–polyacrylamide gel electrophoresis and blotted onto nitrocellulose membranes. Blots were incubated with biotinylated Helix pomatia agglutinin (HPA) that detects O-glycosylated proteins. Lane 1, molecular mass markers; lanes 2 and 3, Phlebotomus sergenti; lanes 4 and 5, P. arabicus; +, preincubation of lectin with 250 mmol/L N-acetyl-d-galactosamine; –, no preincubation. Right, reaction of fluorescein-conjugated HPA with P. arabicus and P. sergenti midgut cells.

Incubation of P. sergenti midgut lysates with HPA showed no reaction, indicating a lack of O-glycosylated proteins (Figure 5). In contrast, an abundant glycoprotein (37–43 kDa) was strongly labeled by HPA in P. arabicus midgut lysates. Controls of P. arabicus midgut lysates incubated with HPA blocked by preincubation with GalNAc showed no reaction, which confirmed the specificity of the lectin reactions in experimental blots (Figure 5). Labeling of midguts with fluorescein-conjugated HPA confirmed the presence of GalNAc-containing glycoproteins in the midguts of P. arabicus. Intensity of labeling in P. sergenti midguts was weaker, which reflected a nonspecific background reaction (Figure 5).

### Comparison of P. sergenti Populations by RAPD and ITS2 Sequencing

**Figure 6:**
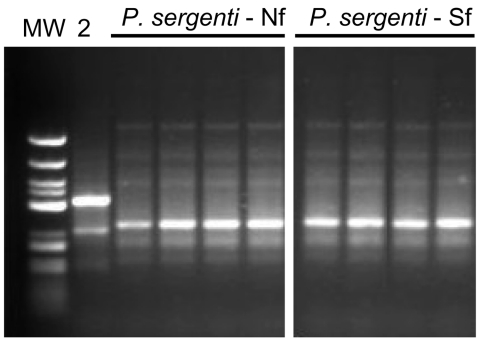
Random amplified polymorphic DNA PCR banding patterns of Phlebotomus sergenti from 2 foci in Galilee, Israel. The PCR was performed with primer OPI 1. Lane MW, molecular mass marker; lane 2, P. sergenti from Turkey. Shown are 4 flies from the northern focus (Nf) and 4 flies from the southern focus (Sf).

Flies from both foci shared the same banding pattern and differed from Turkish P. sergenti (Figure 6). ITS 2 sequences of P. sergenti from both foci were identical with each other and nearly identical (99%) with the ITS 2 sequence of a P. sergenti specimen from the West Bank (GenBank accession no. AF462325) (data not shown).

## Discussion

We have identified 2 emerging foci of CL in which rock hyraxes serve as reservoir hosts of the causative agent L. tropica. Despite their geographic proximity, the 2 foci show fundamental differences with regard to transmission cycles. Parasites and vector species in the southern focus are typical of most Asian zoonotic L. tropica foci, but the northern focus is characterized by antigenically distinct parasites that are transmitted by a newly incriminated sand fly vector.

L. tropica is widely distributed in eastern and northern Africa, the Middle East, and large parts of Asia. A recent study using 21 microsatellite loci showed that L. tropica is a genetically heterogeneous species composed of >80 genotypes. The genetic makeup of this complex suggests a probable African origin, with isolates from the northern focus more related to African isolates than to other strains from the Middle East (*16*).

The major surface molecule of Leishmania promastigotes is LPG, which has been shown to mediate attachment of parasites to the midgut of the sand fly (*8*). LPG of L. tropica from the northern focus is characterized by abundant terminal β-galactose residues on side chains. Conversely, β-galactose residues on LPG side chains of other L. tropica isolates are mostly capped with glucose (*27*). Differences in sugar moieties may have a role in infection of P. sergenti (Figure 4). Although β-galactose residues are present in L. major LPG, strains of L. tropica from the northern focus were not infective to P. papatasi, the natural vector of L. major (M. Svobodova, unpub. data) (*4*).

P. sergenti is probably a species complex, and its component populations show several molecular and morphologic differences (*25*). RAPD-PCR is a powerful tool for estimating genetic variability and was successfully used to compare genetic variation within and between 5 sympatric Phlebotomus species in Spain (*28*). Using the same primer sets, we did not find any differences between P. sergenti flies from the 2 foci (Figure 6). We deduce that populations from both foci are probably freely interbreeding.

P. sergenti is of Palaearctic origin; flies migrated into North Africa during the Miocene era (*29*). Thus, L. tropica and P. sergenti apparently originated in different continents and their geographic overlap probably arose at a later time. P. sergenti, P. (Larroussius) guggisbergi, P. (Paraphlebotomus) saevus, and perhaps P. arabicus are vectors in Africa (*30**,**31*). Since L. tropica variants from both foci develop in P. arabicus, but only the variant from the southern focus completes development in P. sergenti, we postulate that L. tropica was initially transmitted by P. arabicus or another permissive vector such as P. (Adlerius) halepensis (*9*). The more common transmission cycle is a later adaptation to P. sergenti, a dominant, widely distributed phlebotomine species.

Refractoriness of P. sergenti to variants of L. tropica from the northern focus is probably due to the lack of HPA-binding proteins on the luminal surface of midgut epithelium. HPA-binding epitopes are present in permissive vectors such as P. arabicus (Figure 4), P. halepensis (P. Volf, unpub. data), and Lu. longipalpis (*32*). These findings support infections with multiple species of Leishmania (*9**,**33*).

The absence of P. arabicus from the north-facing slopes of the southern foci contrasts dramatically with its predominance in the south-facing slopes of the northern focus. Although a satisfactory explanation for this fact is lacking, such phenomena are not unusual. For example, species richness of insects was much higher in the drier and warmer south-facing slopes of a narrow canyon (100–400 m wide) in Mount Carmel, Israel, than in the north-facing slope of the same canyon (*34*). P. arabicus is widely distributed in Africa and the Arabian peninsula (*17*), and the Galilee focus forms the northern limit of its distribution. Since P. arabicus originates in warmer regions, finding it in warmer, drier, south-facing slopes and not in cooler, shadier north-facing slopes of the hills in Galilee is not surprising (Table 1).

Rock hyraxes in both foci were found infected with L. tropica, and 1 isolate was obtained from an adult male in the northern focus. Although rock hyraxes were suspected reservoir hosts of L. tropica in Africa (*35**,**36*) and have been previously implicated in the northern focus (*4*), this is the first report of a rock hyrax isolate that was identified as L. tropica and shown to be identical to those obtained from humans and sand flies in the same focus (Table 3).

**Figure 7:**
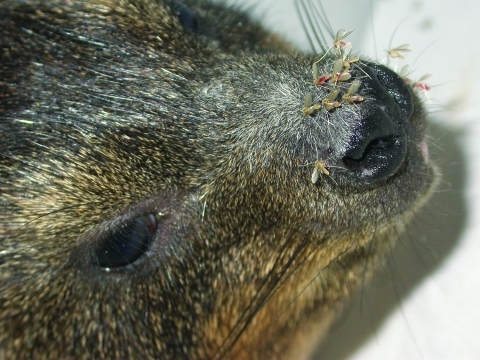
Rock hyrax (Procavia capensis). Sand flies are attracted to these animals and prefer feeding on their snouts.

Rock hyrax populations in many parts of Israel are expanding rapidly and encroaching upon human habitation. They were extremely common in both foci studied, as well as in other L. tropica foci in the region (D. Meir and A. Warburg, unpub. data [4,5], ). In the Galilee foci, rock hyraxes inhabit crevices within boulder mounds that were created when land was cleared for the construction of houses. These artificial caves also afford suitable breeding sites for sand flies. Rock hyraxes are susceptible to L. tropica, and infected rock hyraxes are infective to feeding P. arabicus and P. sergenti. Sand flies are attracted to rock hyraxes and prefer feeding on their snouts (Figure 7) (*37*). This behavior makes them suitable as vectors because L. tropica is usually found in the skin above the nose (R.W. Ashford, unpub. data). Furthermore, as gregarious diurnal mammals, sleeping rock hyraxes are a readily available blood source for night-questing phlebotomine females. Lastly, rock hyraxes live for 9–10 years in the wild (*38*) and constitute an efficient parasite reservoir for infecting sand flies that emerge in the spring after their winter diapause (*39*). These facts indicate that that burgeoning, peridomestic rock hyrax populations are the primary cause of the emergence of CL caused by L. tropica in the region studied (*39**,**40*).
